# Shexiang Tongxin dropping pill for coronary microvascular disease: rationale and design of a multicenter randomized trial with a cardiopulmonary exercise testing primary endpoint and AI-enhanced myocardial contrast echocardiograph

**DOI:** 10.3389/fcvm.2026.1875936

**Published:** 2026-06-18

**Authors:** Meiyi Su, Yuqing Tang, Pengtao Sun, Huan Cen, Shaonan Liu, Xianlun Li, Wei Wang, Lei Wang

**Affiliations:** 1 The Second Clinical College, Guangzhou University of Chinese Medicine, Guangzhou, China; 2 The Second Affiliated Hospital of Guangzhou University of Chinese Medicine, Guangzhou, China; 3 Dongguan Hospital of Traditional Chinese Medicine, Guangzhou University of Chinese Medicine, Dongguan, China; 4 Department of Ultrasonography, The Second Affiliated Hospital of Guangzhou University of Chinese Medicine, Guangzhou, China; 5 China-Japan Friendship Hospital (Institute of Clinical Medical Sciences), Chinese Academy of Medical Sciences & Peking Union Medical College, Beijing, China; 6 Department of Integrative Medicine Cardiology, China-Japan Friendship Hospital, Beijing, China

**Keywords:** artificial intelligence, cardiopulmonary exercise testing, coronary microvascular disease, myocardial contrast echocardiography, randomized controlled trial, shexiang tongxin dropping pill

## Abstract

**Background:**

Coronary microvascular disease (CMVD) is a major cause of angina in patients with ischemia and nonobstructive coronary arteries (INOCA) syndrome. It is characterized by an imbalance between myocardial oxygen supply and demand, leading to reduced exercise tolerance and impaired quality of life. Because of heterogeneous diagnostic approaches and the lack of disease-modifying therapies, CMVD remains underdiagnosed and undertreated. Shexiang Tongxin Dropping Pill (STDP), a traditional Chinese medicine formulation, has shown protective effects on the coronary microvasculature in preclinical and preliminary clinical studies. However, high-quality randomized evidence and objective functional validation are still lacking. This study therefore aims to evaluate the efficacy and safety of STDP in patients with CMVD using cardiopulmonary exercise testing (CPET) as the primary functional outcome and to develop an artificial intelligence (AI)-assisted myocardial contrast echocardiography (MCE) tool to improve CMVD detection and subgroup classification.

**Design:**

This is a prospective, multicenter, randomized, double-blind, placebo-controlled clinical trial with a nested diagnostic design. CMVD will be diagnosed by stress MCE, and eligibility will require evidence of reduced myocardial perfusion or perfusion defects. Eligible patients with CMVD will be randomized at a 1:1 ratio to receive STDP or a matched placebo for 12 weeks. The primary endpoint is the change in peak oxygen uptake (peak VO₂) measured by cardiopulmonary exercise testing (CPET). The secondary outcomes include other CPET indices, angina severity, quality of life, and circulating biomarkers. The nested diagnostic study uses retrospectively and prospectively collected MCE datasets to develop AI-based models for automated myocardial segmentation, perfusion curve analysis, and CMVD classification, with performance assessed against established reference standards.

**Discussion:**

By combining quantitative microvascular imaging, functional exercise assessment, and AI-assisted diagnostics in a single protocol, this study proposes a therapeutic-diagnostic framework for CMVD. This study is expected to provide high-quality randomized evidence for the use of STDP in CMVD and to offer a scalable and objective approach to improve diagnosis, risk stratification, and individualized management in patients with functional coronary ischemia.

**Clinical Trial registration:**

http://itmctr.ccebtcm.org.cn/, International Traditional Medicine Clinical Trial Registry (ITMCTR), TMCTR2025000414.

## Introduction

Coronary microvascular disease (CMVD) is increasingly recognized as a major mechanism of ischemia with nonobstructive coronary arteries (INOCA) (1). CMVD is defined as structural and/or functional abnormalities of the coronary microcirculation that impair myocardial perfusion and lead to recurrent angina, reduced exercise tolerance, and poorer quality of life. CMVD is common in women, people with diabetes, and patients with cardiac syndrome X (2). Its prevalence is difficult to determine because diagnostic pathways vary and functional testing is not standardized. Among patients evaluated for angina, 40%–70% have no obstructive epicardial coronary disease, and approximately 20%–30% of this subgroup meets the diagnostic criteria for CMVD (3). Current guideline-directed care focuses on antianginal therapy and risk factor control (1). These approaches may improve symptoms in some patients, but they do not directly address the underlying microvascular pathology. As a result, recurrent symptoms, limited functional capacity, and residual cardiovascular risk often remain.

Noninvasive imaging and functional assessment may help address these diagnostic and treatment gaps. Stress myocardial contrast echocardiography (MCE) allows real-time quantitative assessment of myocardial microvascular perfusion and provides hemodynamic information related to ischemic physiology (4). Stress MCE yields the stress‒to‒rest myocardial blood flow (MBF) ratio, termed the myocardial blood flow reserve (MBFR), which is a validated index of microvascular vasodilatory capacity (5). When obstructive epicardial coronary disease has been excluded, MBFR is functionally comparable to coronary flow reserve (CFR). Compared with radionuclide imaging and cardiac magnetic resonance, MCE can be performed at the bedside, does not involve ionizing radiation, can be repeated, and is relatively cost-effective. These features support its use in longitudinal evaluation in multicenter studies (6, 7). Along with perfusion imaging, cardiopulmonary exercise testing (CPET) provides an objective and integrated assessment of functional capacity, gas exchange, and overall cardiopulmonary performance (8). In CMVD, impaired microvascular perfusion limits the ability to match myocardial oxygen delivery to metabolic demand during exercise, which contributes to exercise intolerance. CPET captures this limitation through peak oxygen uptake (peak VO_2_), a robust and prognostically relevant measure that links symptoms to physiological impairment and helps distinguish cardiac from noncardiac causes of exercise limitation (9).

Recent advances in artificial intelligence (AI) offer tools for automated myocardial segmentation, kinetic modeling of perfusion curves, and pattern recognition. In this study, AI-assisted analysis will be applied to assess its impact on interobserver variability, detection of subtle perfusion abnormalities, and standardization of perfusion classification. This approach is designed to enhance MCE-based workflows and facilitate standardized analysis for CMVD evaluation.

Shexiang Tongxin Dropping Pill (STDP; National Medical Products Administration approval No. Z20080018) is a proprietary Chinese medicine derived from the classical prescription Zhibaodan and formulated according to the aromatic-warming principle for coronary artery disease. Standardized quality control procedures and chemical fingerprinting have been established (10). Preclinical studies suggest that STDP has anti-inflammatory, endothelial-protective, and cardioprotective effects, partly by reducing macrophage-mediated inflammation, vascular dysfunction, and microvascular obstruction after ischemia–reperfusion injury (11, 12). In clinical settings, preliminary benefits have been reported in patients with coronary slow flow (13). However, its efficacy in nonobstructive coronary artery disease and CMVD remains uncertain because adequately powered and rigorously designed randomized controlled trials with objective physiological and functional endpoints are limited.

Therefore, we designed the STDP-CMVD trial, a hybrid therapeutic-diagnostic study. This study includes a multicenter randomized controlled trial to evaluate the efficacy and safety of STDP and a nested diagnostic study that uses MCE-derived datasets to develop and validate an AI-assisted automated tool for CMVD detection and stratification.

## Study design, methods and analyses

### Study design

**Figure 1:**
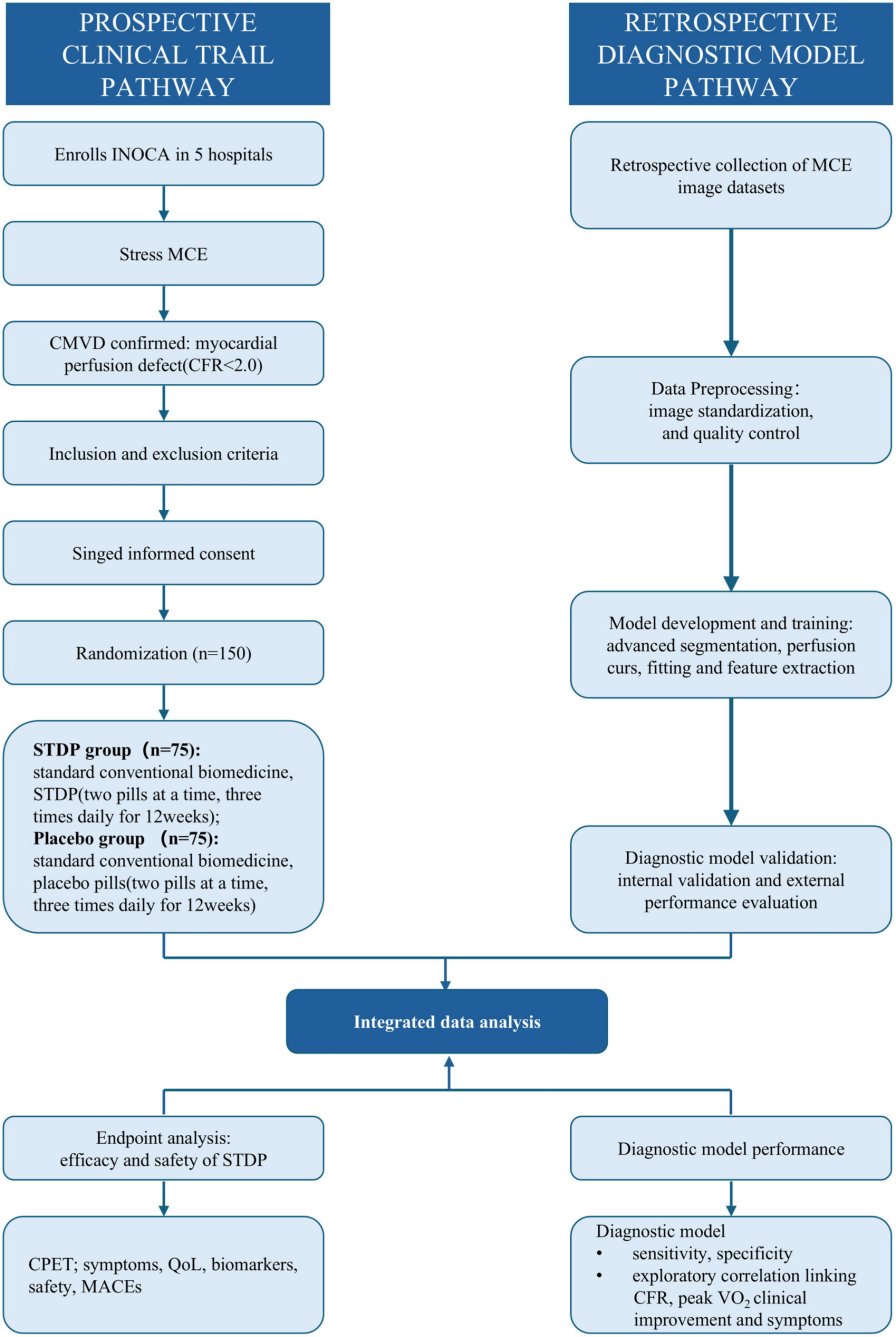
Study design of the STDP-CMVD randomized controlled trial integrating functional and imaging-based assessments. CMVD, coronary microvascular disease; MCE, myocardial contrast echocardiography; CFR, coronary flow reserve; CPET, cardiopulmonary exercise testing.

This is a multicenter, randomized, double-blind, placebo-controlled trial designed to evaluate the efficacy and safety of STDP in patients with CMVD. The diagnosis of CMVD is confirmed by stress MCE during the screening period, with eligibility requiring evidence of reduced myocardial perfusion or perfusion defects (CFR <2.0). After providing written informed consent, eligible participants will be randomized at a 1:1 ratio to receive STDP or a matched placebo in addition to guideline-directed standard therapy for 12 weeks. The study included a screening period of up to 1 week, a 12-week treatment period, and end-of-treatment assessments. The primary endpoint is the change in exercise capacity measured by the peak VO₂ on the CPET. The secondary endpoints include additional CPET indices, angina burden, psychological status, health-related quality of life, and circulating biomarkers. The overall study flowchart is shown in Figure 1.

The study was approved by the Ethics Committee of Guangdong Provincial Hospital of Chinese Medicine (No. BF-2024-250-01). The protocol follows the SPIRIT recommendations, will be conducted in accordance with Good Clinical Practice (GCP), and will be reported in line with the CONSORT. During the study, any protocol changes that may affect study conduct, participant safety, or scientific integrity will be submitted as amendments and approved by the ethics committees at all participating sites before implementation.

### Recruitment

#### Recruitment and study setting

Participants will be screened and enrolled from cardiology departments or chest pain clinics in tertiary hospitals in Beijing and Guangdong Province, including Guangdong Provincial Hospital of Traditional Chinese Medicine (Guangzhou), China–Japan Friendship Hospital (Beijing), Guangdong Provincial People's Hospital (Guangzhou), Nanfang Hospital of Southern Medical University (Guangzhou), Dongguan Hospital of Guangzhou University of Chinese Medicine (Dongguan), and Zhongshan Hospital of Traditional Chinese Medicine (Zhongshan). This setting will allow the recruitment of a demographically diverse population from both northern and southern China. Recruitment will be conducted through investigator referrals and public advertisements, including online platforms and offline posters or flyers placed in outpatient areas, hospital bulletin boards, and community settings.

During prescreening, trained research staff will provide standardized information regarding study objectives, procedures, anticipated benefits, and potential risks. Interested individuals will then undergo clinical evaluation and diagnostic assessment. Eligibility will be assessed independently by two senior physicians with at least 5 years of clinical experience. Eligible candidates who provide written informed consent will be enrolled and randomized via a centralized, web-based randomization system. For individuals who fail screening, basic demographic information and reasons for ineligibility will be recorded. The planned recruitment period is February 28, 2025, to December 31, 2027.

#### Diagnostic criteria

CMVD will be diagnosed according to the Integrated TCM–Western Medicine Guidelines for CMVD (14), with a focus on the microvascular angina (MVA) phenotype. The participants must satisfy all four domains:

1. Symptoms suggestive of myocardial ischemia:
  (a) Typical angina (exertional or at rest), or
  (b) Atypical ischemic equivalents (e.g., dyspnea, interscapular pain).
2. Exclusion of obstructive epicardial coronary artery disease (CAD): Coronary CTA or invasive angiography demonstrating <50% diameter stenosis or stenosis ≥50% and ≤75% with fractional flow reserve (FFR) or quantitative flow ratio (qFR) > 0.80.
3. Objective evidence of myocardial ischemia (≥1 of the following):
  (a) Ischemic ECG changes during spontaneous chest pain episodes;
  (b) stress-induced chest pain and/or ischemic electrocardiographic changes, with or without wall motion abnormalities and/or perfusion defects.
4. MCE revealed reduced myocardial perfusion or perfusion defects.

#### Inclusion criteria

Participants will be eligible if all of the following are met:

1. CMVD was diagnosed according to the above criteria;
2. Angina episodes ≥2 times per week;
3. Age 18–80 years;
4. Willingness and ability to complete the study procedures and follow-up, with written informed consent.

#### Exclusion criteria

Participants will be excluded if any of the following apply:

1. Known hypersensitivity to STDP or its components;
2. Noncardiac causes of chest pain (e.g., gastrointestinal or musculoskeletal);
3. Persistent severe angina (Canadian Cardiovascular Society (CCS) class IV);
4. ST-segment depression was marked with routine activity;
5. Left ventricular ejection fraction (LVEF) <30%, refractory heart failure, or cardiogenic shock;
6. Planned coronary artery bypass grafting (CABG) or percutaneous coronary intervention (PCI) during the study period;
7. Epicardial coronary spasm documented by acetylcholine testing, angiographic spasm, or typical vasospastic features judged clinically;
8. Conditions that may affect CPET interpretation or increase risk, such as chronic obstructive pulmonary disease, poorly controlled asthma, interstitial lung disease, pulmonary embolism, or severe anemia;
9. eGFR <30 mL/min/1.73 m^2^;
10. Significant hepatic disease or ALT/AST >3× upper limit of normal;
11. Serious comorbidities (e.g., malignancy) likely limit survival/adherence;
12. Uncontrolled severe hypertension or clinically significant arrhythmias;
13. Participation in another clinical trial within 30 days or prior to randomization;
14. Pregnant or planned pregnancies during the study period;
15. Any condition judged by investigators to compromise safety or adherence;
16. Severe valvular heart disease or cardiomyopathy;
17. Known allergies to ultrasound contrast agents;
18. Absolute contraindication to pharmacologic stress, such as regadenoson or adenosine.

### Sample size estimation

The primary efficacy endpoint is the change in peak VO₂ after 12 weeks of treatment. The sample size estimation was based on data from the iPOWER study (15), in which the CFVR served as the diagnostic criterion for coronary microvascular dysfunction (CMD). In participants with a CFVR ≥2.0 (non-CMD group, *n* = 22), the median peak VO₂ was 28.6 mL/kg/min (IQR: 22.1–31.7); in those with a CFVR <2.0 (CMD group, *n* = 27), the median peak VO_2_ was 17.3 mL/kg/min (IQR: 15.5–21.3). On the basis of these data, the estimated between-group difference in means was 4.71 mL/kg/min, with a pooled standard deviation of 6.5 mL/kg/min. Using a two-sided superiority design with a significance level *α* = 0.05 and a target power of 90% (1-*β* = 0.90), the required evaluable sample size per group was calculated using the formula for two-independent-sample *t*-test with equal variances:

$$n=2\times {\left(\frac{Z_{1-\alpha /2}+Z_{1-\beta }}{\Delta /\sigma }\right)}^{2}$$

Where $\;Z_{1-\alpha /2}$ = 1.96, $Z_{1-\beta }$  = 1.282, and *δ* = 4.71, *σ* = 6.5. This given *n* = 41.5 per group, rounded up to 42 evaluable subjects per group (total 84). The calculation was performed using PASS 2023 (NCSS, LLC, Kaysville, UT, USA), which confirmed that 42 subjects per group achieve 90.68% power under the same assumptions.

To account for an anticipated 15% attrition rate (dropout or loss to follow-up), the sample size was inflated using the formula:

$$n^{\prime }=\frac{n}{1\text{-}\mathrm{DR}}$$

where DR is the dropout rate. With *n* = 42 and *DR* = 0.15, *n'*=42/0.85 = 49.41, rounded up to 50 participants per group. Thus, the final target sample size is 100 participants in total (50 per group).

### Randomization and allocation concealment

The participants will be randomized at a 1:1 ratio through a centralized web-based system managed by the Clinical Trial Management Office of Guangdong Provincial Hospital of Traditional Chinese Medicine. A site-stratified block randomization sequence will be generated via SAS (v9.4; SAS Institute Inc.). Allocation concealment will be ensured through centralized drug coding. Center identifiers, randomization numbers, subject identification numbers, and medication codes will be maintained by an independent statistical unit.

### Blinding and emergency unblinding

This was a double-blind trial. The master randomization list and blinding code will be generated and maintained by an independent statistician with restricted access. Investigators, participants, outcome assessors, and site staff will remain blinded until database lock. The study drug and placebo will be identical in appearance, packaging, and labeling. Emergency unblinding will be allowed only when needed for the clinical management of a serious adverse event. Unblinding will be performed through a 24 hour interactive response system and will be documented, including the date, reason, and responsible personnel. It will also be reported to the study monitor within 24 h. Planned unblinding will take place only after completion of the final visit and database lock.

### Intervention and concomitant medication management

#### Study intervention

The participants will receive one of the following treatments:

- **STDP group:** guideline-directed standard therapy plus STDP (two pills, three times daily) for 12 weeks;
- **The placebo group received** guideline-directed standard therapy plus a matched placebo (two pills, three times daily) for 12 weeks.

Standard therapy will follow the EAPCI Expert Consensus on the INOCA (16). This treatment may include antianginal drugs, such as beta-blockers, calcium channel blockers, and long-acting nitrates, as well as lipid-lowering therapy, antiplatelet agents when indicated, and risk factor management. Concomitant medications for hypertension, diabetes, or dyslipidemia will be allowed according to clinical needs and must be documented. To reduce confounding, guideline-directed therapies should be kept at stable doses whenever clinically feasible. Any treatment changes and the reasons for these changes will be recorded.

#### Prohibited medications

The use of other Chinese herbal medicines with Qi-tonifying or blood-activating effects will not be allowed during the trial.

#### Compliance monitoring

Adherence will be assessed at each visit via pill counts and participant medication diaries. Electronic monitoring may also be used when feasible. Adherence will be calculated as (doses taken ÷ doses prescribed) × 100%. An adherence rate of at least 80% was considered acceptable. Participants with poor adherence will receive counseling. Persistent nonadherence may lead to the discontinuation of study treatment. Adherence data will be recorded in the eCRF.

### Stress MCE protocol and interpretation

Stress MCE will be performed at baseline via contrast-specific low-mechanical-index imaging (target mechanical index 0.05–0.20) on a standardized platform (EPIQ 7C, Philips Healthcare) with an S5-1 transducer (2.0–5.0 MHz). A sulfur hexafluoride microbubble contrast agent (SonoVue, Bracco Suisse S.A.) will be administered intravenously according to the manufacturer's instructions to achieve homogeneous myocardial opacification. A constant infusion of approximately 1 mL/min will be maintained during rest and stress image acquisition, followed by a saline flush when needed.

Pharmacologic stress will be induced with 0.4 mg of intravenous regadenoson administered over 10 s (*T* = 0). Image acquisition will focus on the hyperaemic window (*T* = 1.5–4 min). Standard apical 4-, 2-, and 3-chamber views will be obtained at rest and during steady-state hyperemia. Destruction–replenishment imaging uses a brief high-mechanical-index flash (approximately 0.8–2.0), followed by replenishment cine recording for at least 10–15 s at a frame rate of at least 25 frames/s to allow curve fitting. The participants will be asked to avoid caffeine or xanthine-containing products for at least 12 h before testing. Continuous electrocardiographic and noninvasive blood pressure monitoring will be performed throughout the procedure.

All image acquisition will be performed by trained echocardiographers blinded to treatment allocation. DICOM data will be archived and transferred to a core laboratory.

#### Qualitative assessment

Two independent expert readers (≥10 years of experience), blinded to the clinical data and treatment allocation, evaluated the perfusion patterns. Disagreements were resolved by consensus or by a third reader. Perfusion abnormalities are classified as perfusion delay, perfusion reduction, or perfusion defects according to core laboratory criteria.

#### Quantitative assessment

Offline analysis will be performed via iMCE software (Narnar, China). The left ventricle was segmented according to the American Heart Association (AHA) 17-segment model. Standardized regions of interest (5 × 5 mm) will be placed within the myocardium, with motion tracking and manual adjustment when needed. The time‒intensity curves were fitted with the following equation:

$$y(t)\;=\;A\;\times \;(1\text{-}e^{\wedge \;}(\text{-}\beta t))\;+\;B$$

In this model, A represents microvascular blood volume, *β* represents microvascular flow velocity, and A×*β* estimates MBF (17). The CFR is calculated as the ratio of the stress MBF to the remaining MBF at the segmental level. The global CFR is obtained by averaging the segmental values. Interobserver and intraobserver variability will be assessed in a random 10% sample.

### CPET protocol and interpretation

The CPET will be performed at baseline and at week 12 according to the AHA and EACPR/AHA scientific statements (18, 19). A symptom-limited incremental protocol will be used on an upright cycle ergometer (Schiller CS200 ERGO) with breath-by-breath gas analysis (Brilliance/SDS-200), and the data will be averaged over 15 sec intervals.

Participants will be instructed to withhold medications that may substantially affect heart rate or blood pressure, such as beta-blockers, calcium channel blockers, and nitrates, for 24 h before testing when clinically safe. They were also instructed to fast and avoid smoking or caffeine for at least 2 h before the test. The protocol will include 2 min of rest, 3 min of warm-up at 20 W, and then incremental loading starting at 25 W with an increase of 25 W every 2 min until voluntary exhaustion or a termination criterion is reached. This was followed by active recovery at 20 W for 3 min and passive recovery for 3 min. Continuous 12-lead electrocardiographic monitoring will be performed, and blood pressure will be measured every 2 min. A peak respiratory exchange ratio (RER) >1.1 will be used as a criterion supporting maximal effort (19, 20). Tests will be stopped for chest pain, significant ST-segment changes, abnormal blood pressure responses, or serious arrhythmias.

The main CPET parameters will include peak VO₂ (mL/kg/min and % predicted), oxygen pulse, *Δ*VO_2_/*Δ*work rate slope, heart rate reserve, and 1 min heart rate recovery (21). The ventilatory anaerobic threshold will be determined by the V-slope method with manual confirmation (22). All tests will be performed by a trained team via a standardized protocol.

### Outcomes

#### Primary outcomes

The primary outcome will be assessed from baseline to week 12 as the change in peak VO₂ measured by the CPET. This primary endpoint provides a comprehensive assessment of functional capacity, integrating both cardiac and peripheral contributions to exercise limitation, and serves as a well-established prognostic marker in patients with cardiovascular disease.

#### Secondary outcomes

The secondary outcomes will be assessed at baseline and week 12 and will include the following:

- **Biomarkers:** lipid profile, homocysteine (HCY), high-sensitivity C-reactive protein (hs-CRP);
- **CPET parameters:** additional functional indices as described above;
- **Symptoms and patient-reported outcomes:** Seattle Angina Questionnaire (SAQ) score, Chinese medicine symptom scoring scale, and visual analog scale (VAS);
- **Psychological status:** Patient Health Questionnaire-9 (PHQ-9) and Generalized Anxiety Disorder-7 (GAD-7);
- **Quality of life:** EuroQol Five-Dimension scale (EQ-5D).

All scale-based assessments will be independently completed by two physicians with at least 5 years of clinical experience, and any disagreements will be resolved by consensus.

### Safety assessment and adverse event reporting

Safety will be assessed by recording all adverse events (AEs), serious adverse events (SAEs), and predefined major adverse cardiovascular events (MACEs). Any treatment discontinuation or modification related to an adverse event will be documented. Laboratory tests, including hematology, liver function, and renal function, as well as vital signs and 12-lead electrocardiography, will be performed at baseline and week 12, with additional assessments as clinically indicated. Adverse event severity will be graded according to CTCAE version 5.0. Potential MACEs will be reviewed by an independent clinical endpoint committee.

### Follow-up, discontinuation, withdrawal, and data management

Eligible participants will be identified on the basis of stress MCE findings during the screening period, with confirmation of CMVD requiring evidence of reduced myocardial perfusion or perfusion defects. Following enrollment, participants will attend onsite visits at week 0 (baseline), week 8 (±7 days), and week 12 (±7 days). Comprehensive efficacy assessments, including the CPET, laboratory tests and safety evaluations, will be conducted at both the baseline visit and the week 12 visit. The week-8 visit will focus on adherence, review of concomitant medication, symptom and patient-reported outcome assessment, and interim safety monitoring (Table 1). Stress MCE is performed only at screening for diagnostic confirmation and is not repeated during follow-up.

**Table 1:** Schedule of assessments and data collection time points for the hybrid study.

Period	Screening period	Pretreatment	Treatment period	Treatment period	Follow-up period
	−1 week	0 week	8 weeks	12 weeks	16 weeks
Baseline information					
Informed consent	√				
Eligibility screen	√				
Demographics and medical history	√				
Physical examination and vital Signs	√			√	
Randomization					
Diagnostic confirmation					
Stress MCE	√				
Outcome measurements					
CPET	√			√	
Echocardiography (Conventional Parameters) and ECG	√				
Laboratory tests(lipid profile, HCY, hs-CRP)	√			√	
SAQ	√		√	√	
Health questionnaires (PHQ-9, GAD-7)	√			√	
Chinese medicine symptom scoring scale	√			√	
Safety assessments					
Liver function	√			√	
Renal function	√			√	
Adverse events			√	√	√
MACEs			√	√	√
Others					
Concomitant medication record	√	√	√	√	
Evaluate compliance			√	√	

HCY, homocysteine; hs-CRP, high-sensitivity C-reactive protein; SAQ, seattle angina questionnaire; PHQ-9, health questionnaire depression scale; GAD-7, generalized anxiety disorder scale; ECG, electrocardiogram; MCE, myocardial contrast echocardiography; CPET, cardiopulmonary exercise testing; MACEs, major adverse cardiovascular events.

“√” represents the need for implementation at this point in time.

Participants may discontinue study treatment because of voluntary withdrawal, pregnancy, hypersensitivity reactions, or adverse events related to the study drug and requiring treatment cessation. When feasible, study staff will attempt to collect end-of-treatment outcome data. For participants who drop out or are lost to follow-up, repeated contact attempts by telephone, written notice, or other approved methods will be made and documented. All the data collected before discontinuation are retained for auditing. The most recent available assessments, including primary endpoint data when available, will be handled according to the prespecified statistical analysis plan.

All study data will be entered into an electronic case report form (eCRF) via the Weiyun Clinical Research Integrated Platform (v3.0; http://we.ttdoc.cn/sp). Double data entry and programmed edit checks will be used to ensure data quality. Audit trials and role-based access control will also be applied.

### Data collection and management

Before trial initiation, all study personnel, including investigators, coordinators, sonographers, CPET technicians, and data managers, will receive standardized training on the protocol, standard operating procedures (SOPs), and good clinical practice (GCP). To minimize inter-center variability, all sites will adhere to the same acquisition protocol. Centralized training and periodic quality audits will be conducted. CPET calibration and analysis will be centrally reviewed. Statistical models will include site as a random effect to account for potential clustering. The training will focus on harmonized stress MCE acquisition, including contrast administration, imaging settings, destruction–replenishment sequences, and timing under regadenoson; standardized CPET procedures, including calibration, incremental protocols, termination criteria, and quality indicators such as the respiratory exchange ratio (RER); and consistent documentation of endpoint-related variables to ensure comparability across study sites.

All study data will be entered into an electronic case report form (eCRF) system with role-based access control. Double data entry will be performed by two independent staff members, and any discrepancies will be resolved by source data verification. Built-in range checks and programmed logic rules will be applied to key variables—including MCE-derived diagnostic confirmation (CFR <2.0), CPET parameters (peak VO₂ and quality indicators), patient-reported outcome scores, visit windows, and medication changes—to minimize transcription errors and ensure data completeness.

To maintain data integrity in this multicenter study, qualified monitors will perform periodic risk-based quality control reviews, including onsite monitoring, remote verification, and regular communication with site teams. Monitoring will assess data completeness, timeliness, logical consistency, and agreement with source documents. Queries are issued through the eCRF system, tracked until resolution, and documented in the audit trail.

Imaging and physiologic data will undergo centralized quality control. All MCE DICOM files will be anonymized at each site, transferred securely to a blinded core laboratory, and analyzed according to prespecified rules. A random 10% sample will be reread to assess interobserver and intraobserver variability. CPET raw files, including calibration logs, peak effort criteria such as RER, and protocol adherence, will be archived and centrally reviewed for quality. For the nested AI study, imaging datasets are deidentified and stored separately from clinical identifiers, and the linkage keys are retained only at the coordinating center.

All study data will be stored in an encrypted electronic environment with restricted access. Personally identifiable information and imaging data receive additional protection through encryption and limited access. Full audit trials will be maintained. Data handling will comply with the Declaration of Helsinki, applicable ethical requirements, and relevant data protection regulations to protect participant privacy and data security.

### Data monitoring committee

Given the low-risk profile of the interventions and the lack of planned interim analyses, a formal data monitoring committee (DMC) will not be convened. Safety oversight will be provided by the sponsor's quality assurance unit through periodic risk-based monitoring, with serious adverse events reviewed promptly. An independent external safety advisor may be consulted if clinically important safety concerns arise.

### Dissemination policy

Trial results will be submitted for publication in a peer-reviewed journal regardless of outcome direction. The participants will receive a lay-summary of findings. Authorship will follow ICMJE guidelines; no professional writers will be used. The full protocol, anonymized participant-level dataset, and statistical code will be available upon reasonable request to the corresponding author.

### Data access

Access to the final trial dataset will be limited to the principal investigators, study coordinators, and independent statistician. No contractual agreements restrict investigator access. De-identified data will be shared with external researchers upon reasonable request after publication, subject to approval by the study steering committee and applicable data protection regulations.

### Statistical analysis

All analyses will be prespecified. The primary analysis will be conducted in the intention-to-treat (ITT) population, including all randomized participants analyzed according to their assigned group. A per-protocol (PP) analysis will be performed as a sensitivity analysis.

Baseline characteristics will be summarized via descriptive statistics. Continuous variables are presented as the means ± SDs if approximately normally distributed or medians (IQRs) otherwise; categorical variables are reported as *n* (%). Normality will be assessed via graphical methods and formal tests (e.g., Shapiro-Wilk).

#### Primary endpoints

The primary endpoint is the change in peak VO_2_ measured by CPET from baseline to week 12. The primary analysis will be performed via an analysis of covariance (ANCOVA) model, with the treatment group as a fixed effect and the corresponding baseline value as a covariate. The study center will be included either as a fixed effect or as a random effect in a mixed-effects model, depending on model fit and the distribution of participants across centers. The results are reported as adjusted mean differences with 95% confidence intervals and two-sided *P* values.

#### Secondary outcomes

Continuous secondary outcomes—including laboratory parameters, patient-reported outcome scores, and CPET indices other than peak VO_2_—will be analyzed as changes from baseline via ANCOVA with baseline adjustment. If distributional assumptions are violated, rank-based ANCOVA or nonparametric methods (e.g., the Mann–Whitney *U*-test) will be applied. Categorical outcomes will be compared via the chi-square test or Fisher's exact test, as appropriate. Exploratory analyses will assess correlations between changes in peak VO_2_ and improvements in symptoms or quality of life (e.g., SAQ scores) via Pearson or Spearman coefficients. Safety outcomes, including adverse events, major adverse cardiovascular events, and changes in liver and renal function, will be summarized descriptively and compared between groups where applicable.

#### Missing data

Missing primary endpoint data at week 12 will be handled via multiple imputation under the missing-at-random assumption. Sensitivity analyses will include complete-case analysis and other prespecified approaches when needed, such as pattern-mixture models or conservative imputation methods.

All the statistical tests will be two-sided. The overall two-sided significance level for the primary endpoints will be set at *α* = 0.05 after adjustment for multiplicity. Analyses will be performed via SPSS (v26.0) and/or validated statistical software (e.g., SAS/R) as specified in the SAP.

#### Medication adherence analysis

Medication adherence will be assessed by structured interviews, medication diaries, and pill counts based on returned containers at each visit. Adherence will be calculated as (doses taken ÷ doses prescribed) ×100% and categorized as good (≥80%), moderate (50%–79%), or poor (<50%). Adherence summaries will be presented to the treatment group. Protocol-defined thresholds are used to guide counseling and reinforcement. Adherence will also be considered in sensitivity analyses, including per-protocol analyses excluding major protocol deviations and exploratory dose‒response analyses when appropriate. All adherence data will be recorded in the eCRF with audit trials.

### Nested diagnostic study: AI model development and validation

**Figure 2:**
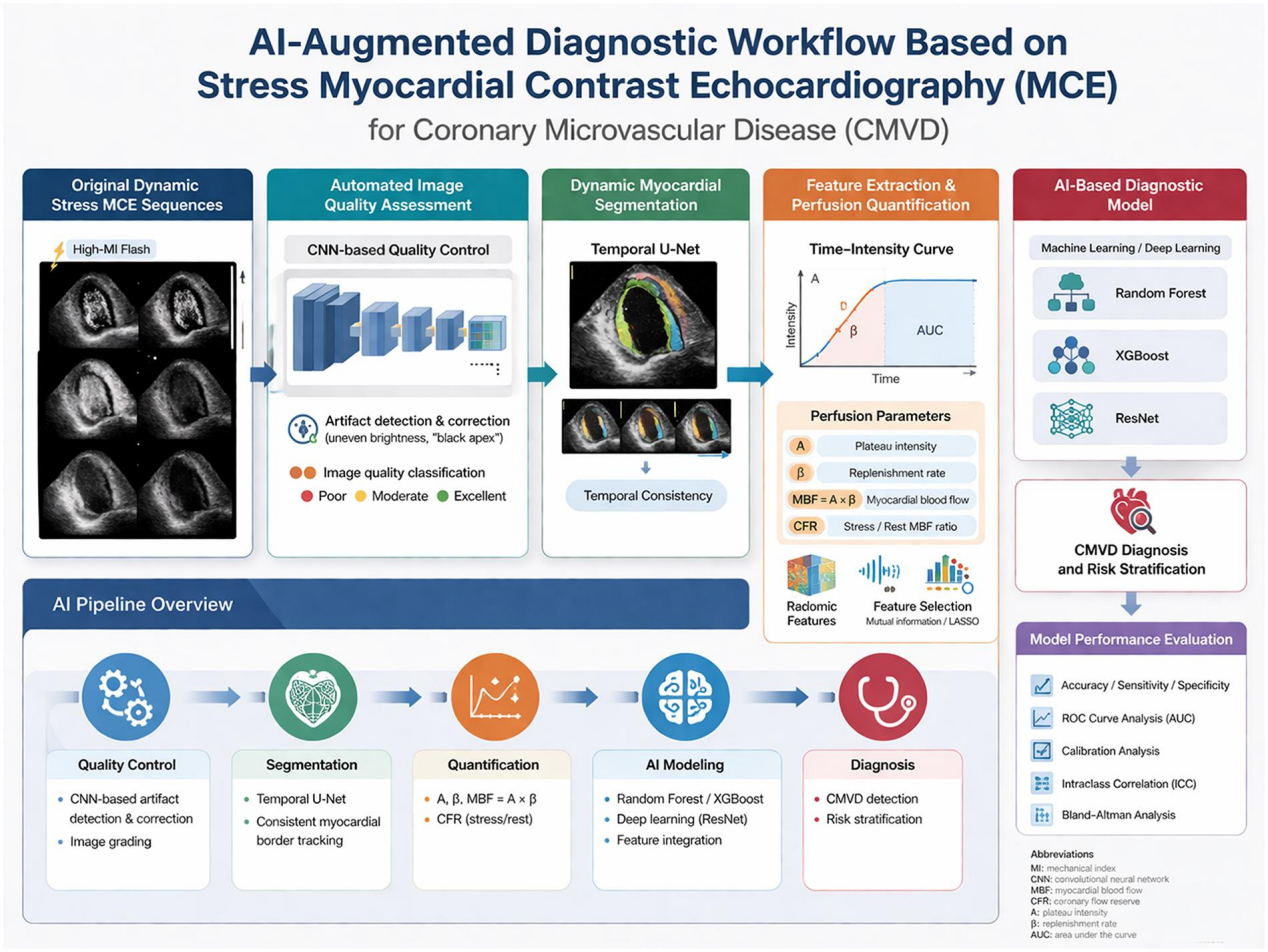
AI-augmented diagnostic workflow for coronary microvascular disease based on stress MCE. Dynamic stress MCE sequences acquired via high–mechanical index (high-MI) flash are first processed by a CNN-based module for image quality assessment, enabling artifact detection and classification. High-quality images are subsequently analyzed via a temporal deep learning model to achieve consistent myocardial segmentation across the cardiac cycle. The quantitative perfusion parameters, including the plateau intensity (A), replenishment rate (*β*), myocardial blood flow (MBF = A × *β*), and CFR, are derived from time–intensity curves. These features, together with radiomic descriptors, are integrated into machine learning models (e.g., random forest, XGBoost, and ResNet) for CMVD diagnosis and risk stratification. Model performance is evaluated via discrimination, calibration, and agreement metrics. MCE, myocardial contrast echocardiography; MI, mechanical index; CNN, convolutional neural network; MBF, myocardial blood flow; CFR, coronary flow reserve.

We will retrospectively collect dynamic stress MCE flash-replenishment sequences from participating centers, de-identify them, and transfer them to a central imaging core laboratory. To minimize interscanner and intersite variation while preserving replenishment kinetics, we apply a standardized preprocessing pipeline that includes speckle denoising with motion stabilization, contrast normalization with intensity scaling, frame alignment with temporal smoothing, and image resizing with pixel-level standardization. Two expert echocardiographers will assign quality labels to build a supervised image quality control module. A convolutional neural network (CNN) will then perform automated image quality classification and artifact detection, such as uneven illumination, attenuation (black apex), and motion blur. We will exclude low-quality frames according to predefined rules and apply artifact correction when feasible. Expert frame-by-frame annotations of endocardial and epicardial borders will serve as the reference to develop a temporally consistent segmentation model (e.g., temporal U-Net or ConvLSTM-U-Net) for stable myocardial tracking across frames. We will evaluate model performance using the Dice coefficient, Hausdorff distance, and temporal consistency metrics. From the segmented myocardium, we will generate pixel-level and segment-level time-intensity curves and fit them for perfusion quantification to derive A, *β*, MBF (A  ×  *β*), and area under the curve (AUC). When paired rest-stress data are available, we will calculate reserve indices, including *β* reserve and A  ×  *β* reserve/CFR. We will also extract radiomic texture features and acoustic features to describe spatial heterogeneity. We will perform feature selection using mutual information and LASSO to reduce redundancy and improve robustness. We will develop CMVD classification models mainly with random forest and XGBoost, and optimize hyperparameters via cross-validation. We will evaluate model performance on separate training, validation, and independent test datasets. External validation will be performed using an independent dataset collected from one participating site that was not used for model training or internal validation. The model's generalizability will be assessed by evaluating its performance on this separate dataset. The functional reference standards will include PET-derived flow reserve or expert-validated iMCE-CFR. Performance metrics will comprise accuracy, sensitivity, specificity, F1 score, and area under the receiver operating characteristic curve (AUC). We will use ROC analysis to assess discrimination, calibration curves to assess agreement between predicted and observed probabilities, and Bland-Altman analysis together with intraclass correlation coefficients to assess agreement between the AI-derived quantitative indices and iMCE outputs. Figure 2 shows the AI-based diagnostic workflow for coronary microvascular disease based on stress MCE.

### Recruitment status

The trial is currently recruiting. As of June 12, 2025, one participant had been enrolled at Guangdong Provincial People's Hospital.

## Discussion

CMVD is characterized by structural and/or functional abnormalities of the coronary microcirculation in the absence of obstructive epicardial coronary artery disease. Although these patients do not have flow-limiting stenosis, they often experience a high burden of angina, reduced quality of life, and increased healthcare use, which creates an ongoing clinical and socioeconomic challenge (23, 24). A definite diagnosis usually requires functional assessment after obstructive epicardial disease has been excluded. However, currently available techniques, including invasive coronary function testing, cardiac magnetic resonance (CMR), and positron emission tomography (PET), are often limited to specialized centers because of their technical complexity and cost. This contributes to the underdiagnosis of CMVD in routine practice. Current treatment strategies are mainly empirical and are often adapted from guidelines for obstructive coronary artery disease rather than being directed at the specific vasomotor, inflammatory, and metabolic mechanisms involved in CMVD. Although early studies suggest that multitarget agents such as STDP may improve microvascular function through anti-inflammatory and vasoregulatory effects, strong evidence from well-designed randomized controlled trials is still limited (25). These unmet needs form the basis for the present study, which combines therapeutic evaluation with diagnostic development to improve CMVD management.

Compared with previous studies in CMVD, the present trial has several distinguishing methodological features. The CorMicA trial demonstrated the clinical value of stratified medicine guided by invasive coronary function testing (CFR, IMR, and acetylcholine provocation) in patients with INOCA (26). The WISE study and ILIAS registry established the prognostic significance of a reduced CFR (<2.0) in symptomatic patients without obstructive coronary artery disease (27, 28). However, these studies were either observational or used invasive diagnostic procedures. In contrast, our study is a randomized, placebo-controlled trial that uses non-invasive stress MCE to confirm CMVD (CFR <2.0) and evaluates functional improvement using CPET-derived peak VO₂ as the primary endpoint. Furthermore, we are developing an AI-based diagnostic model using MCE datasets to automate myocardial segmentation and perfusion analysis. This integrated strategy bridges the diagnostic gap in CMVD while providing high-level evidence for targeted therapy.

Recent progress in noninvasive cardiac imaging has expanded the tools for assessing coronary microvascular function. Stress MCE is a clinically feasible, quantitative, and radiation-free technique that can be performed at the bedside using widely available ultrasound equipment, making it suitable for longitudinal multicenter studies. Compared with PET and CMR, MCE offers practical advantages of lower cost, greater accessibility, and repeatability. A CFR threshold below 2.0 on stress MCE is widely accepted as a diagnostic criterion for CMVD. A reduced CFR reflects a mismatch between myocardial oxygen supply and metabolic demand during stress, which may clinically manifest as reduced exercise tolerance (29). These features support stress MCE as an effective tool for diagnosing CMVD and identifying patients who may benefit from targeted interventions.

CPET is the gold standard for evaluating functional capacity in patients with cardiovascular disease (30). In CMVD, impaired coronary microvascular vasodilation leads to a mismatch between myocardial oxygen supply and demand during physical exertion, resulting in reduced exercise tolerance (21). CPET objectively quantifies this limitation through key gas exchange parameters. Peak VO_2_ is a strong predictor of cardiovascular morbidity and mortality and has been shown to correlate with coronary microvascular function in symptomatic populations (8). In the iPOWER study, women with confirmed CMD (CFVR <2.0) had markedly lower peak VO_2_ values than asymptomatic controls with normal microvascular function, independent of age and cardiovascular risk factors (15). Other CPET-derived indices, such as oxygen pulse, ventilatory efficiency (VE/VCO_2_ slope), and heart rate recovery, provide further insight into cardiovascular and autonomic adaptation to exercise, and may reveal subclinical functional impairment even in the absence of overt ischemia (31). Given its ability to capture the physiological consequences of microvascular dysfunction, CPET is well suited for assessing treatment efficacy in CMVD trials (32). Improvement in peak VO_2_ after intervention may reflect not only better myocardial oxygen delivery but also meaningful gains in daily functional capacity and quality of life (33).

Mechanistic studies suggest that STDP may have pleiotropic effects on several pathways involved in CMVD. It may suppress M1 macrophage overactivation through the Dectin-1/Syk/IRF5 signaling pathway, thereby reducing inflammation and endothelial dysfunction (11). It may also downregulate ALOX12 expression, which may help reduce microvascular spasm and obstruction and preserve myocardial microcirculation (12). In addition, STDP may activate the PI3K/Akt/mTORC1 pathway, promote macrophage polarization toward a repair-related phenotype, and support functional angiogenesis, which may help restore the structure and function of the coronary microvasculature (34). The multicomponent and multitarget features of STDP are consistent with the complex pathophysiology of CMVD and support a broader therapeutic approach. Although previous clinical studies have reported improvements in microvascular angina and endothelial function with STDP (35), most of those studies relied on subjective symptom scores or invasive measures. By using the stress MCE as a diagnostic tool and the CPET as the primary efficacy assessment, the present trial may provide a direct and objective evaluation of functional improvement in patients with CMVD. Concurrently, the nested development of an AI-based MCE diagnostic model is intended to standardize perfusion analysis. Automated image quality control, dynamic myocardial segmentation, and perfusion curve quantification aim to reduce operator dependence and improve reproducibility, potentially facilitating broader application of functional microvascular assessment. Combining a therapeutic trial with diagnostic development in one protocol is a key methodological feature of this study. Progress in CMVD care depends on improvements in both treatment and patient identification. A validated therapy may increase the need for accurate diagnostic tools, whereas better diagnostic methods may improve the selection of patients most likely to benefit from targeted interventions such as STDP.

This study has several limitations. First, the 12-week treatment period limits evaluation of long-term outcomes, especially MACEs, which need longer follow-up. Second, the study is underpowered to detect differences in MACEs. Third, despite our efforts to standardize procedures across centers, residual inter-center variability exists; we will adjust for it in the analysis. Fourth, stress MCE's diagnostic accuracy depends on image quality and operator experience. A core laboratory and standardized protocols reduce, but do not eliminate, this limitation. Also, MCE-derived CFR does not capture all features of microvascular dysfunction, such as endothelial-dependent vasomotion. Fifth, the AI model requires external validation in other populations and settings before broad use.

Traditional Chinese medicine(TCM) trials carry inherent bias. The placebo matches the active drug in appearance and packaging, but patients' prior beliefs about TCM may affect their expectations. We did not assess placebo credibility or expectation bias. To minimize confounding, we kept guideline-directed antianginal therapy stable and documented any changes. Still, we cannot fully exclude cultural or contextual effects.

Nevertheless, we expect this study to provide pilot data, establish a reproducible methodological framework, and inform the design of larger confirmatory trials. Overall, this study aims to offer a more integrated management approach for patients with CMVD.

## Trial status

This study is currently ongoing. Ethical approval for the initial protocol was obtained from the Ethics Committee of Guangdong Provincial Hospital of Chinese Medicine on November 15, 2024 (Approval No. BF2024-250-01), and the trial was registered on the International Traditional Medicine Clinical Trial Registry (ITMCTR) on February 26, 2025 (Registration No. TMCTR2025000414). The first participant was enrolled on June 12, 2025, at Guangdong Provincial People's Hospital.

Following expert panel review and protocol refinement, the study design was optimized to enhance feasibility across participating centers. The stress MCE was retained as the core diagnostic tool for confirming CMVD (CFR <2.0) during screening, whereas the CPET was designated the primary efficacy endpoint to assess functional improvement. This adjustment reflects the complementary roles of microvascular imaging and functional assessment in evaluating treatment response and ensures consistent implementation across all centers while preserving the objective nature of the primary outcome. The revised protocol (current version) received ethical approval on November 20, 2025 (Approval No. BF-2025-250-04). The study is expected to be completed in December 2027.

## Protocol version

20250915, Version 4.0.

## Supplementary material

Supplementary File 1 Biological specimen processing.

## References

[1] Vrints C Andreotti F Koskinas KC Rossello X Adamo M Ainslie J. 2024 ESC guidelines for the management of chronic coronary syndromes. Eur Heart J. (2024) 45(36):3415–537. 10.1093/eurheartj/ehae177 39210710

[2] Bradley C Berry C. Definition and epidemiology of coronary microvascular disease. J Nucl Cardiol. (2022) 29(4):1763–75. 10.1007/s12350-022-02974-x 35534718 PMC9345825

[3] Parlati ALM Nardi E Sucato V Madaudo C Leo G Rajah T. ANOCA, INOCA, MINOCA: the new frontier of coronary syndromes. J Cardiovasc Dev Dis. (2025) 12(2):64. 10.3390/jcdd12020064 39997498 PMC11856364

[4] Gvinianidze L Toulemonde M Hampson R Huang B Bioh G Wakefield LA. Ultrafast myocardial contrast echocardiography for the assessment of coronary artery disease: first in-human study. Circ Cardiovasc Imaging. (2024) 17(10):e17267. 10.1161/CIRCIMAGING.124.017267 PMC11483224 39355914

[5] Taqueti VR Di Carli MF. Coronary microvascular disease pathogenic mechanisms and therapeutic options: jACC state-of-the-art review. J Am Coll Cardiol. (2018) 72(21):2625–41. 10.1016/j.jacc.2018.09.042 30466521 PMC6296779

[6] Crea F Montone RA Rinaldi R. Pathophysiology of coronary microvascular dysfunction. Circ J. (2022) 86(9):1319–28. 10.1253/circj.CJ-21-0848 34759123

[7] Huang W Morello M Gholson BA Lindner JR. Precision medicine in ischemic heart disease through point-of-care myocardial contrast echocardiography. JACC Cardiovasc Imaging. (2024) 17(10):1246–51. 10.1016/j.jcmg.2024.07.022 39243234

[8] de Boer E Petrache I Mohning MP. Cardiopulmonary exercise testing. JAMA. (2022) 327(13):1284–5. 10.1001/jama.2022.2037 35266955 PMC13166173

[9] Kim SR Cho D Kim M Park SM. Rationale and study design of differences in cardiopulmonary exercise capacity according to coronary microvascular dysfunction and body composition in patients with suspected heart failure with preserved ejection fraction. Int J Heart Fail. (2021) 3(4):237–43. 10.36628/ijhf.2021.0029 36262558 PMC9536684

[10] Chen D Lin S Xu W Huang M Chu J Xiao F. Qualitative and quantitative analysis of the Major constituents in Shexiang Tongxin dropping pill by HPLC-Q-TOF-MS/MS and UPLC-QqQ-MS/MS. Molecules. (2015) 20(10):18597–619. 10.3390/molecules201018597 26473821 PMC6331871

[11] Cui L Liu Y Hu Y Dong J Deng Q Jiao B. Shexiang Tongxin dropping pill alleviates M1 macrophage polarization-induced inflammation and endothelial dysfunction to reduce coronary microvascular dysfunction via the dectin-1/syk/IRF5 pathway. J Ethnopharmacol. (2023) 316:116742. 10.1016/j.jep.2023.116742 37290736

[12] Wu Y Lin Y Liu B Ma J Xiang Y Wang Y. Shexiang Tongxin dropping pill ameliorates microvascular obstruction via downregulating ALOX12 after myocardial ischemia-reperfusion. Int J Cardiol. (2024) 416:132481. 10.1016/j.ijcard.2024.132481 39179033

[13] Wang S Chu L Xu Z Zhou HL Chen JF Ning HF. Effect of Shexiang Tongxin dropping pills () on the immediate blood flow of patients with coronary slow flow. Chin J Integr Med. (2019) 25(5):360–5. 10.1007/s11655-018-2559-4 29915906

[14] Project Group for the Clinical Practice Guideline on Integrated Traditional Chinese and Western Medicine for Coronary Microvascular Disease. Coronary microvascular disease: a clinical practice guideline for integrated traditional Chinese and western medicine. Chin J Integr Trad West Med. (2023) 43(9):1029–39.

[15] Bechsgaard DF Hove JD Suhrs HE Bové KB Shahriari P Gustafsson I. Women with coronary microvascular dysfunction and no obstructive coronary artery disease have reduced exercise capacity. Int J Cardiol. (2019) 293:1–9. 10.1016/j.ijcard.2019.07.048 31345648

[16] Kunadian V Chieffo A Camici PG Berry C Escaned J Maas AHEM. An EAPCI expert consensus document on ischaemia with non-obstructive coronary arteries in collaboration with European society of cardiology working group on coronary pathophysiology & microcirculation endorsed by coronary vasomotor disorders international study group. Eur Heart J. (2020) 41(37):3504–20. 10.1093/eurheartj/ehaa503 32626906 PMC7577516

[17] Porter TR Mulvagh SL Abdelmoneim SS Becher H Belcik JT Bierig M. Clinical applications of ultrasonic enhancing agents in echocardiography: 2018 American society of echocardiography guidelines update. J Am Soc Echocardiogr. (2018) 31(3):241–74. 10.1016/j.echo.2017.11.013 29502588

[18] Balady GJ Arena R Sietsema K Myers J Coke L Fletcher GF. Clinician’s guide to cardiopulmonary exercise testing in adults. Circulation. (2010) 122(2):191–225. 10.1161/CIR.0b013e3181e52e69 20585013

[19] Guazzi M Adams V Conraads V Halle M Mezzani A Vanhees L. EACPR/AHA scientific statement. Clinical recommendations for cardiopulmonary exercise testing data assessment in specific patient populations. Circulation. (2012) 126(18):2261–74. 10.1161/CIR.0b013e31826fb946 22952317 PMC4777325

[20] Glaab T Taube C. Practical guide to cardiopulmonary exercise testing in adults. Respir Res. (2022) 23(1):9. 10.1186/s12931-021-01895-6 35022059 PMC8754079

[21] Dores H Mendes M Abreu A Durazzo A Rodrigues C Vilela E. Cardiopulmonary exercise testing in clinical practice: principles, applications, and basic interpretation. Rev Port Cardiol. (2024) 43(9):525–36. 10.1016/j.repc.2024.01.005 38583860

[22] Brazile TL Levine BD Shafer KM. Cardiopulmonary exercise Testing. NEJM Evid. (2025) 4(2):EVIDra2400390. 10.1056/EVIDra2400390 39873542

[23] Shimokawa H Suda A Takahashi J Berry C Camici PG Crea F. Clinical characteristics and prognosis of patients with microvascular angina: an international and prospective cohort study by the coronary vasomotor disorders international study (COVADIS) group. Eur Heart J. (2021) 42(44):4592–600. 10.1093/eurheartj/ehab282 34038937 PMC8633728

[24] Wang ZJ Zhang LL Elmariah S Han HY Zhou YJ. Prevalence and prognosis of nonobstructive coronary artery disease in patients undergoing coronary angiography or coronary computed tomography angiography: a meta-analysis. Mayo Clin Proc. (2017) 92(3):329–46. 10.1016/j.mayocp.2016.11.016 28259226

[25] Zhu L Yang Y Huang Y Xie HK Luo Y Li C. Shexiang Tongxin dropping pills protect against ischemic stroke-induced cerebral microvascular dysfunction via suppressing TXNIP/NLRP3 signaling pathway. J Ethnopharmacol. (2024) 322:117567. 10.1016/j.jep.2023.117567 38122909

[26] Ford TJ Stanley B Good R McEntegart M Watkins S Eteiba H. Stratified medical therapy using invasive coronary function testing in angina: the CorMicA trial. J Am Coll Cardiol. (2018) 72(23 Pt A):2841–55. 10.1016/j.jacc.2018.09.006 30266608

[27] Albadri A Bairey Merz CN Johnson BD Wei J Mehta PK Cook-Wiens G. Impact of abnormal coronary reactivity on long-term clinical outcomes in women. J Am Coll Cardiol. (2019) 73(6):684–93. 10.1016/j.jacc.2018.11.040 30765035 PMC6383781

[28] Boerhout CKM De Waard GA Lee JM Mejia-Renteria H Lee SH Jung JH. Prognostic value of structural and functional coronary microvascular dysfunction in patients with non-obstructive coronary artery disease; from the multicentre international ILIAS registry. EuroIntervention. (2022) 18(9):719–28. 10.4244/EIJ-D-22-00043 35694826 PMC10241297

[29] Padro T Manfrini O Bugiardini R Canty J Cenko E De Luca G. ESC Working group on coronary pathophysiology and microcirculation position paper on ‘coronary microvascular dysfunction in cardiovascular disease’. Cardiovasc Res. (2020) 116(4):741–55. 10.1093/cvr/cvaa003 32034397 PMC7825482

[30] Parasuraman S Schwarz K Singh S Abraham D Garg D Frenneaux MP. Cardiopulmonary exercise test in myocardial ischemia detection. Future Cardiol. (2020) 16(2):113–21. 10.2217/fca-2019-0022 32081024

[31] Gakidi A Kotoulas S Pataka A Pitsiou G Boutou A. Cardiopulmonary exercise testing in chronic diseases. Front Sports Act Living. (2025) 7:1598498. 10.3389/fspor.2025.1598498 40260420 PMC12009904

[32] Bjarnason-Wehrens B Schmidt T Schwaab B. Cardiopulmonary exercise testing for exercise prescription in cardiac rehabilitation. Herzschrittmacherther Elektrophysiol. (2023) 34(1):26–32. 10.1007/s00399-022-00921-4 36720723

[33] Karlsson P Lind L Michaelsson K Malinovschi A. Cardiopulmonary exercise testing and body composition. ERJ Open Res. (2024) 10(3):00970–2023. 10.1183/23120541.00970-2023 38887678 PMC11181054

[34] Lu X Yao J Li C Cui L Liu Y Liu X. Shexiang Tongxin dropping pills promote macrophage polarization-induced angiogenesis against coronary microvascular dysfunction via PI3K/akt/mTORC1 pathway. Front Pharmacol. (2022) 13:840521. 10.3389/fphar.2022.840521 35401214 PMC8984141

[35] Zhao Y Xing Y Zou K Jiang WD Du TH Chen B. Shexiang Tongxin dropping pill improves stable angina patients with phlegm-heat and blood-stasis syndrome: a multicenter, randomized, double-blind, placebo-controlled trial. Chin J Integr Med. (2025) 31(8):685–93. 10.1007/s11655-025-4014-7 40512367

