# Supplementary material for: Shexiang Tongxin dropping pill for coronary microvascular disease: rationale and design of a multicenter randomized trial with a cardiopulmonary exercise testing primary endpoint and AI-enhanced myocardial contrast echocardiograph
Source: Front Cardiovasc Med. 2026 Jun 18;13:1875936. doi: 10.3389/fcvm.2026.1875936 (PMC13322804; doi:10.3389/fcvm.2026.1875936)
Supplement: Supplementary File 1 — Biological specimen processing. [file Table1.docx]

**Biological specimens**

After obtaining written informed consent, 4 mL of venous blood will be collected from each participant into an anticoagulant vacuum tube containing ethylenediaminetetraacetic acid (EDTA). The blood sample will be centrifuged at 3000 rpm for 10 minutes at 4°C. Following centrifugation, the supernatant (plasma) will be transferred to a sterile centrifuge tube on ice and subjected to a second centrifugation at 3500 rpm for 10 minutes at 4°C. The resulting plasma will be aliquoted into labeled 2 mL cryovials (0.3–0.5 mL per vial) and stored at –80°C. All biospecimens will be maintained in the Biological Resources Center of Guangdong Provincial Hospital of Chinese Medicine in accordance with its standard operating procedures. Specimens will be retained for five years after study completion, after which they will be destroyed.
